# Molecular mechanisms of aging and anti-aging strategies

**DOI:** 10.1186/s12964-024-01663-1

**Published:** 2024-05-24

**Authors:** Yumeng Li, Xutong Tian, Juyue Luo, Tongtong Bao, Shujin Wang, Xin Wu

**Affiliations:** 1 Tianjin Institute of Industrial Biotechnology, Chinese Academy of Sciences; National Center of Technology Innovation for Synthetic Biology, Tianjin, China; 2 Institute of Life Sciences, Chongqing Medical University, Chongqing, China

**Keywords:** Aging, Aging triggers, Synthetic, Senolytic, Anti-aging strategies

## Abstract

Aging is a complex and multifaceted process involving a variety of interrelated molecular mechanisms and cellular systems. Phenotypically, the biological aging process is accompanied by a gradual loss of cellular function and the systemic deterioration of multiple tissues, resulting in susceptibility to aging-related diseases. Emerging evidence suggests that aging is closely associated with telomere attrition, DNA damage, mitochondrial dysfunction, loss of nicotinamide adenine dinucleotide levels, impaired macro-autophagy, stem cell exhaustion, inflammation, loss of protein balance, deregulated nutrient sensing, altered intercellular communication, and dysbiosis. These age-related changes may be alleviated by intervention strategies, such as calorie restriction, improved sleep quality, enhanced physical activity, and targeted longevity genes. In this review, we summarise the key historical progress in the exploration of important causes of aging and anti-aging strategies in recent decades, which provides a basis for further understanding of the reversibility of aging phenotypes, the application prospect of synthetic biotechnology in anti-aging therapy is also prospected.

## Background

Aging will be a major social problems worldwide in the coming decades [1–3]. During the aging process, the body tissues and organs of the older people undergo functional decline or deterioration, thus increasing their susceptibility to age-related diseases and shortening their healthy life span, which has brought enormous financial pressure to countries worldwide in terms of pension, medical expenses, and health care [4–6]. Therefore, exploring the biological nature of aging, searching for safe and effective intervention strategies to positively regulate health status, and prolonging the healthy lifespan of the aging population are important for reducing the global pension burden and promoting healthy aging.

**Fig. 1:**
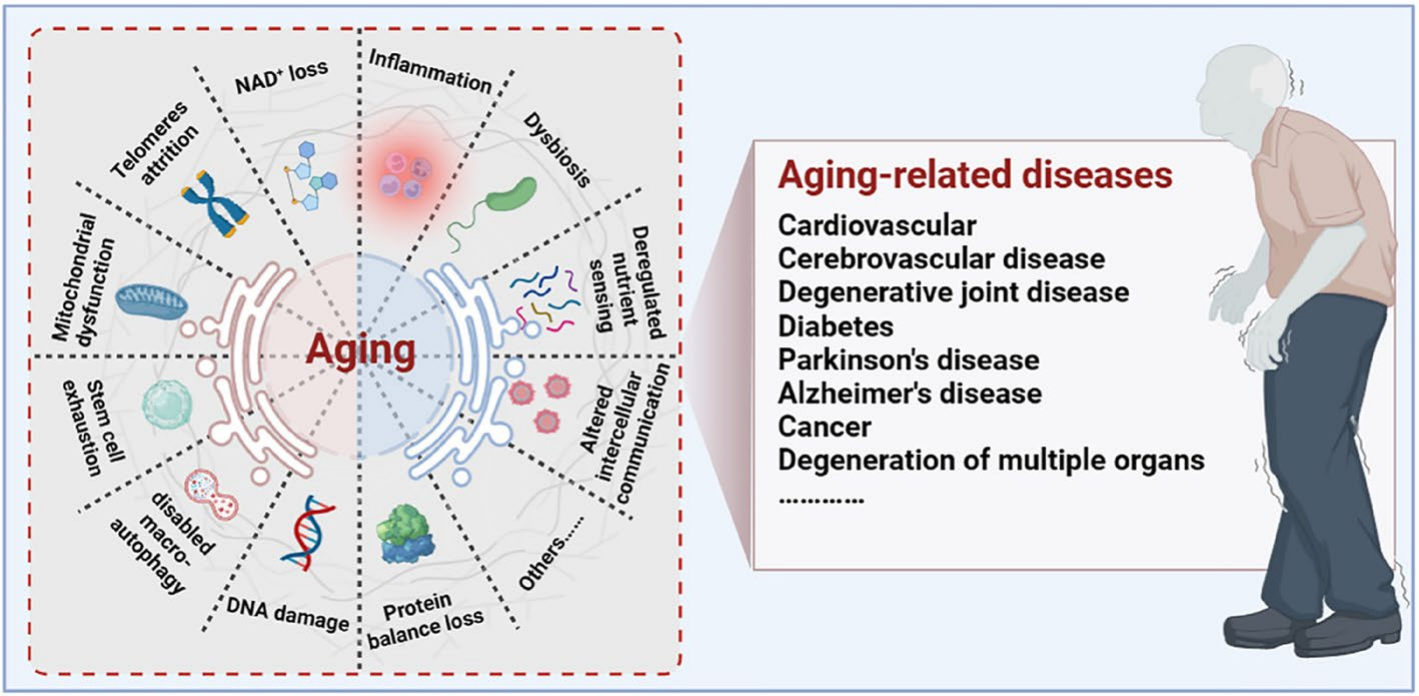
Aging drivers and age-related diseases. Major physiological features of aging include NAD^+^ loss, telomeres attrition, mitochondrial dysfunction, stem cell exhaustion, disabled macro-autophagy, DNA damage, protein balance loss, inflammation, dysbiosis, deregulated nutrient sensing, and altered cellular communication. These physiological characteristics of aging are primitive, antagonistic, and integrated, and their interaction promotes aging. When aging reaches a certain threshold, organ and tissue function continues to deteriorate, which increases the incidence and mortality of aging-related diseases, including cardiovascular, cerebrovascular, degenerative joint disease, diabetes, Parkinson’s disease, Alzheimer’s disease, and cancer

Aging is a progressive degenerative state that can be physiological and pathological [7–9] (Fig. 1). Physiological aging is observed in across many species, and is a degenerative process that occurs after maturation, including telomere attrition [10, 11], DNA damage [12, 13], mitochondrial dysfunction [14, 15], loss of nicotinamide adenine dinucleotide (NAD^+^) levels [16, 17], impaired macro-autophagy [18, 19], stem cell exhaustion, inflammation [20, 21], loss of protein balance [22], deregulated nutrient-sensing [23], altered intercellular communication [24–26] and dysbiosis [27, 28], thereby leading to systemic functional decline. Importantly, these changes are decentralised and interactive, not independent of each other. Pathological aging includes the senile pathological aging changes, which are caused by various external factors, such as cardiovascular disease [29], cerebrovascular disease [30], degenerative joint disease [31, 32], diabetes [33], Parkinson’s disease [34, 35], Alzheimer’s disease [36], cancer [37–39], and degeneration of multiple organ functions. These aging-induced cellular physiological and pathological changes can reflect the underlying nutrient sensing, intercellular communication, protein stabilisation, epigenetics, and molecular abnormalities in DNA damage repair, leading to genomic instability and damage. Further understanding of the different molecular mechanisms involved in the aging process is of great importance for preventing aging and prolonging the lifespan.

In recent years, a large number of animal and clinical experiments have been conducted to study factors that induce aging, such as morphological and pathological changes and functional decline of the aging organism. Indeed, some differences between biological and chronological age reflect the validity of age-accelerated or deceleration procedures, which are well-known biomarkers of the aging process. Researchers have gradually expanded from traditional methods of measuring aging (including maximal energy expenditure at the respiratory, sensory, psychomotor, and cognitive levels) to modern biotechnological methods, such as genomics, epigenomics, transcriptomics, proteomics, and metabolomics. These techniques may have implications for assessing the spatiotemporal patterns of health degradation and effectiveness of anti-aging strategies.

Briefly, aging is a complex process, and its characteristics are interdependent. Each of these factors should be considered as an entry point for future exploration of the aging process and the development of novel life extensions. Here, we review the history and current state of aging research and summarise the characteristics of aging and the mechanisms promoting aging. In addition, we review different types of aging mechanisms and their corresponding anti-aging strategies. This knowledge can guide the design of preventive and therapeutic strategies to delay aging and age-related diseases and extend human health and longevity.

## Potential triggers and molecular mechanisms of aging

Aging is a complex result of many biological processes, and many key factors trigger aging, such as DNA damage, telomere dysregulation, mitochondrial dysregulation, NAD^+^ loss, autophagy disorders, and stem cell exhaustion. Here, we summarise the main causes and underlying molecular mechanisms contributing to the aging process.

### Aging and DNA damage

DNA damage is a major internal factor that leads to genomic instability, epigenetic changes, protein stress, impaired mitochondrial function, and telomere dysfunction [12]. The continuous accumulation of DNA-damaged cells triggers cell death and senescence, ultimately leading to chronic inflammation, loss of function, atrophy, and disease in cells and tissues [40].

#### Molecular mechanisms of DNA damage

Genomic instability manifests as permanent and transmissible changes in DNA sequence [13, 41]. DNA damage caused by an inherently unstable genome includes spontaneous deamination, hydrolysis, and many other chemical changes such as different types of breaks, changes in base positions, gaps, DNA-protein cross-links, and other subtle chemical modifications [12, 42]. Abnormal DNA structures (e.g. G-quadruplexes, R-loops, and persistent single-stranded regions), as well as abnormal intermediates in DNA transactions (e.g. stalled transcription, replication, and recombination complexes), are considered phenotypes of DNA damage [13]. Genomic mutations caused by DNA instability adversely affect cellular functions and are major causes of cancer and genetic diseases. However, DNA instability is also the most important substrate in the evolution of species [43, 44]. DNA integrity is maintained by the continuous repair of highly complex DNA repair and DNA damage response (DDR) systems that counteract the time-dependent erosion and destruction of genetic DNA information. Progressive telomere shortening is another major contributor to DNA damage that accelerates the aging process [45].

DNA damage is the major driver of age-related epigenetic changes. The epigenome, which includes DNA methylation and histone modifications, is unstable throughout the life cycle of somatic cells [46]. DNA damage leads to persistent chromatin changes that enrich aging-enhancing DNA fragments (DNA-scars) in senescent cells [47]. Persistent DNA damage and repair-related cellular physiological effects may leave epigenetic marks, resulting in epigenetic heterogeneity among cells. Transcription appears to change considerably more in senescent cells than in young cells. Thus, DDR may be a major cause of epigenetic changes that impair gene expression control, leading to somatic heterogeneity and a time-dependent decline in overall function.

#### Relationship between DNA damage and aging

**Fig. 2:**
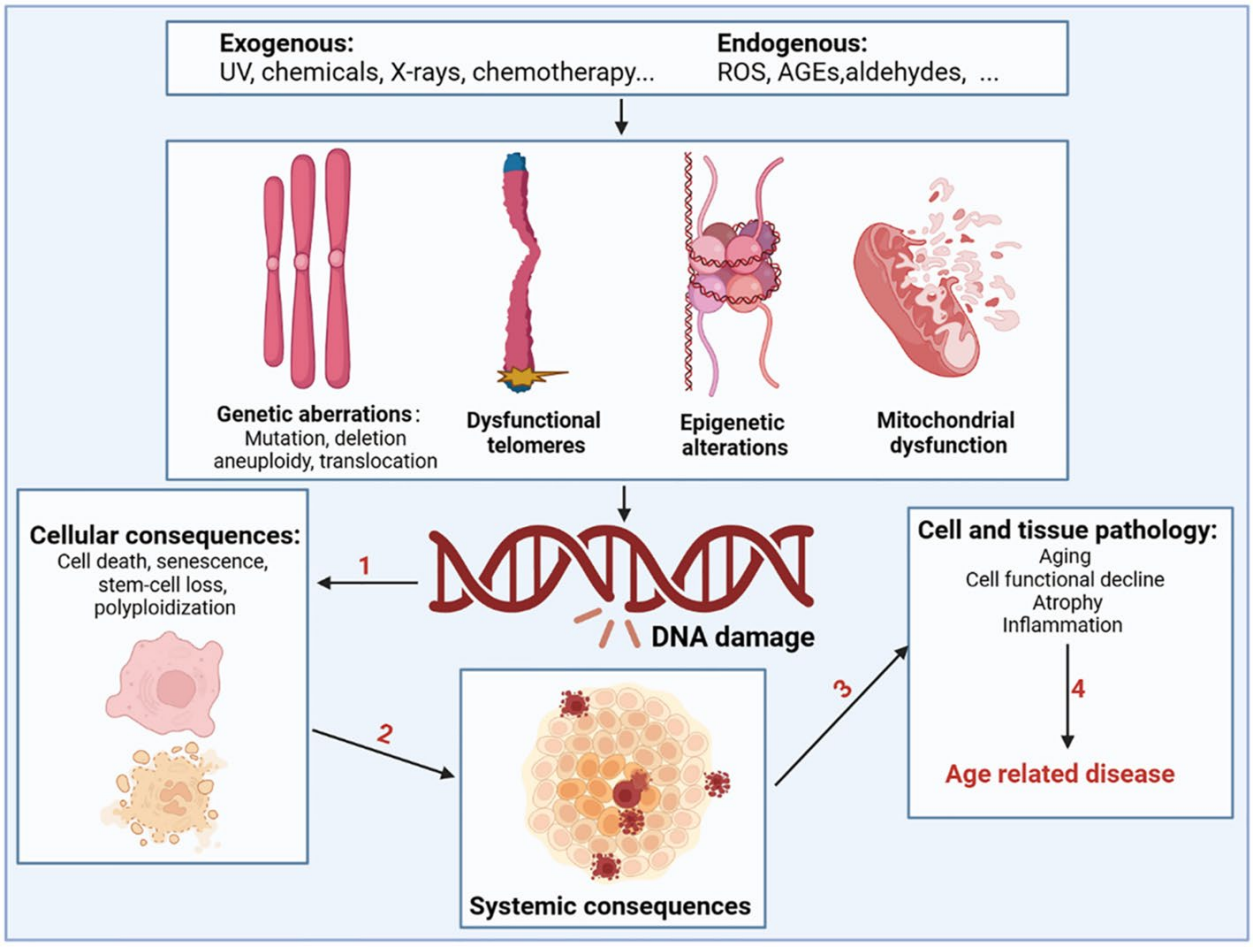
Drivers of DNA damage and the resulting systemic consequences. The nuclear and mitochondrial genomes are constantly exposed to exogenous substances (such as ultraviolet and X-rays, chemicals in food, water, and air), endogenous substances such as ROS, advanced glycation end products (AGEs), and aldehydes; this results in genetic abnormalities, including mutation, deletion, aneuploidy, translocation, dysfunctional telomeres, epigenetic alterations, and mitochondrial dysfunction. DNA damage and DNA damage response caused by the above factors can shock molecular processes and alter cell fate, such as cell death, senescence, and systemic breakdown of repair functions, eventually leading to the loss of cell and organ function and promoting the occurrence and development of age-related diseases

During aging, numerous exogenous and endogenous genotoxins, photoaging, and mechanical stress in tissues continuously induce DNA damage (Fig. 2). Approximately 10^5^ DNA damage events occur in mammalian cells every day, although most of the DNA damage is effectively excised or repaired. Notably, a small portion escapes the DNA damage detection and repair system, subsequently resulting in failure to repair or repair errors [48]. Many studies using mammalian models have confirmed an inextricable link between DNA damage and aging [49–52]. As aging progresses, the DNA repair capacity gradually declines, and the increased molecular phenotype of genomic instability becomes the main marker of aging. Markers of DNA damage are found in patients with age-related diseases such as cardiovascular disease [53], Alzheimer’s disease [54], and cancer [55], suggesting that DNA damage is directly related to the incidence of these diseases. Patients with genetic or acquired defects in DNA repair proteins also exhibit features of premature aging and that differences in the location of the defect in the DNA repair system can lead to premature aging in different organs [56]. Specifically, RecQ helicase plays an important role in DNA recombination, replication, repair, and telomere maintenance, and its mutation may increase the incidence of Werner, Bloom, and Rothmund-Thomson syndromes [57]. Global genome nucleotide excision repair deficiency leads to a thousand-fold increase in skin cancer susceptibility and may accelerate neurodegeneration [58]. Impaired transcription-coupled repair mechanisms can cause typical age-related pathologies, such as neurodegeneration, osteoporosis, and atherosclerosis [59]. Hutchinson-Gilford progeria is associated with nuclear genome instability, defects in DNA double-strand break repair leading to telangiectasia and Nijmegen break syndrome, and defects in DNA cross-linking repair leading to anaemia [60]. In addition, DNA damage caused by mitochondrial defects is another underlying factor in a class of progressive diseases that affect multiple organs.

Overall, defects in the DNA damage repair system directly leads to the continued accumulation of genomic mutations, which underlie many segmental forms of premature aging in humans, suggesting a close link between genome integrity and aging. Although considerable progress has been achieved in the study of the mechanistic connection between DNA damage and aging, there are still many issues to be further explore the specific molecular mechanisms by which DNA damage affects diseases in older people. Therefore, fundamentally addressing the aging process and combating age-related diseases are important for exploring the relationship between DNA damage and anti-aging effects.

### Aging and telomere attrition

**Fig. 3:**
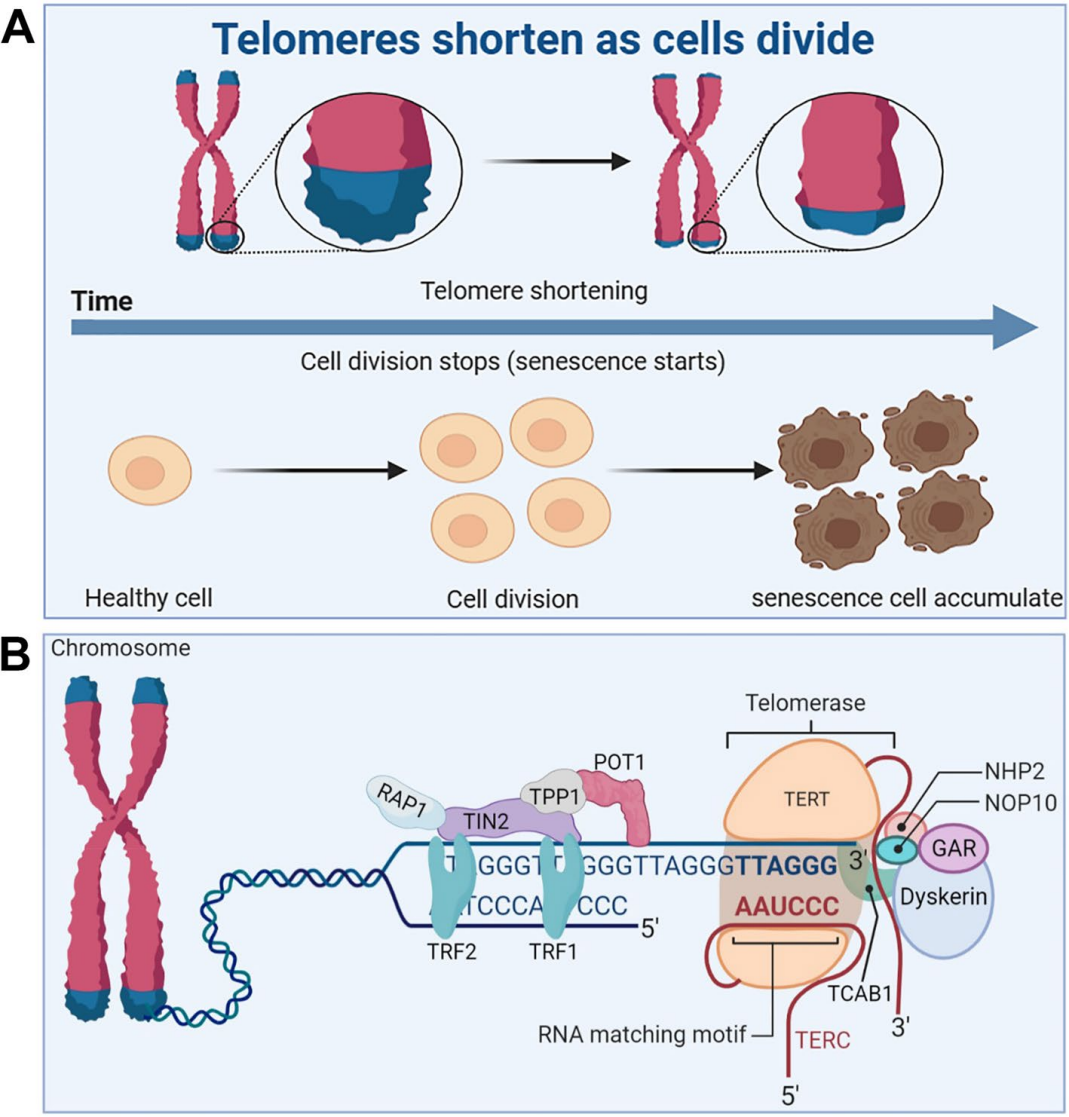
Telomere and telomerase structure, and their relationship with cell senescence. **A** Telomeres shorten during cell division, leading to accumulation of senescent cells. **B** The structure of the telomere-telomerase complex. TERT, telomerase reverse transcriptase; TERC, telomerase RNA component; NOP10, nucleolar protein family A, member 3; NHP2, nucleolar protein family A, member 2; GAR, nucleolar protein family A; TIN2, TERF1-interacting nuclear factor 2; TPP1, telomere protection protein 1; TRF1, telomeric repeat binding factor 1; TRF2, telomeric repeat binding factor 2; POT1, protection of telomeres 1; RAP1, TERF2-interacting protein. The telomere diagram is derived from “biorender”

Telomeres are small stretches of DNA-protein complexes present at the ends of linear chromosomes in eukaryotic cells, which maintain chromosomal integrity, control the cell division cycle, and are essential for an organism’s healthy life span and reproduction [61]. As early as the 1960s, a scientist named Leonardo Hayflick discovered that cultured human fibroblasts had limited and reproducible replication capacity and were governed by cell-autonomous mechanisms [62, 63]. Even if the cold stops the cell division, once the temperature rises again, the cells will continue to divide before freezing, until 50 times after the cessation of division. Heverick realized that cells have a deep-seated internal mechanism that controls the number of times they divide [64]. In the 1970s, Olovnikov [65] and Watson [66] discovered the “end duplication problem” by looking at asymmetries in linear DNA replication and predicting that each cell division results in chromosomal DNA at the ends of the lagging strands loss, eventually leading to the gradual shortening of chromosomes. Limited telomere length reserve is an obstacle to cell proliferation and viability, and the loss of telomere function is closely associated with age-related functional decline and increased incidence of disease [67] (Fig. 3A).

#### Telomere and telomerase structure

Telomere end protection is evolutionarily highly conserved from lower to higher multicellular organisms [68]. Structurally, telomeres consist of repeating nucleotide sequences 3’-[TTAGGG]-5’ in tandem, ranging from a few to tens of bases, terminated at the 3’ end by a single strand of guanine-rich nucleotides of 75 to 300 nt, forming a “cap structure” (Fig. 3B). Telomeres are covered by a special protein called the shelterin complex, which is a multimer of six protein subunits (TRF1, TRF2, TPP1, POT1, TIN2, and RAP1) that work together to protect the chromosomes and regulate telomere length [68]. Telomeres and shelterin complexes form a sophisticated higher-order structure that protects DNA repair programmes from fusing ends by mediating non-homologous end-joining of telomeric DNA through double-stranded DNA break detection, ultimately involved in the capping, protection, and regulation of telomeres [69]. Correspondingly, mutations in these six protein components can disrupt the shelterin-telomere complex, resulting in terminal fusion and premature senescence. Specifically, telomere maintenance is inseparable from normal expression of TRF1 [70, 71]. TRF1 deletion induces telomeric DNA to form a fragile site phenotype, whereas TRF1 overexpression impairs telomerase binding to telomere ends, eventually resulting in telomere shortening [72, 73]. TRF2 folds telomeric DNA into T-loops, inhibits the ataxia telangiectasia mutated-dependent DDR at chromosome ends, and suppresses end-to-end chromosome fusion and canonical homologous end joining [74]. In addition, TIN2 plays a connecting role in the shelterin complex and forms bridges between different shelterin proteins [75]. TIN2 mutations do not interfere with the spatial structure of other shelterin components on telomeres; however, the TIN2-R282H mutation activates telomeric DNA damage signalling, which results in telomere instability associated with telomerase activity, eventually leading to a premature cellular senescence phenotype [76]. Uncontrolled POT1 impairs telomerase binding to telomere ends, resulting in shortened telomeres [77]. TPP1 interacts with telomerase reverse transcriptase (TERT) to recruit telomerase and its loss elicits a robust telomeric DNA damage response [78]. Rap1 is a key telomere-capping protein that prevents non-homologous end joining and telomere fusion, and its overexpression causes histone loss and accelerates cellular senescence [79, 80]. Overall, the biological functional integrity of telomeres depends on the interaction of telomeres and the shelterin complex, which together regulate telomere length and the cell life cycle. It should be noted that normally shortened telomeres alone do not drive senescence (biology) if telomeres become so short that they are perceived as double-stranded DNA breaks, then these telomeres will recruit the DDR and induce the cells into a normal apoptotic or senescence program.

Telomerase is a riboprotease composed of two basic subunits: TERT and telomerase RNA component (TERC) [81]. The H/ACA domain of Cajal body protein 1 in TERC binds to telomerase to form telomerase Cajal body protein 1, which catalyzes telomerase activity and transports telomerase to the ends of telomeres [82]. In addition, multiple core protein components, including dyskerin, NHP2, NOP10, and GAR1, are essential for the normal catalytic function of telomerase [83]. Normally, telomerase is abundantly expressed in undifferentiated stem [67] and progenitor cells of germ cells [84], the skin, intestine [85], haematopoietic system [82], hair bulge [86], and testis [7]. Nevertheless, it is extremely low or undetectable in differentiated adult cells, such as neuroblasts [87], fibroblasts [88], cardiomyocytes [89], and sperm cells [90]. In the germ line and in some stem cells, telomerase can compensate for this loss of telomere duplication, which decreases with cell division [91]. Telomerase is silent during the early development of most somatic cells, limiting the number of cell divisions until the telomeres become very short [92]. The pathogenicity of telomere shortening during aging is a characteristic antagonistic pleiotropic effect. On the one hand, cells with telomere dysfunction are prone to genome instability and may become cancer cells. On the other hand, the normal replicative shortening of telomeres can restrict unrestricted cell proliferation and induce cell apoptosis or senescence, thus preventing the formation of tumors. Robinson et al. found a way to help telomeres maintain their length, a technique known as alternative lengthening of telomeres (ALT) [93]. In osteosarcoma and bread cancer cell lines, the potential relationship between telomere lengthening and inhibition of tumor growth is cleverly orchestrated in cell lines that maintain telomere length by the ALT [94]. It helps that tumors can be suppressed even when telomeres are lengthened.

Maintenance of adequate telomere length in normal cells requires intact telomere structure and highly sophisticated regulation of telomerase [95]. However, each associated protein in the telomere and telomerase complexes is susceptible to uncontrollable factors in the tissue microenvironment [96]. However, there is still some scientific debate regarding how the telomerase complex is sensed, expressed, and recruited to telomere ends for functional regulation to determine the role of telomeres and telomerases in the pathogenesis of systemic aging and degenerative diseases. Recently, telomere dysfunction has been described as a molecular feature of senescent cells, and the loss of telomere function is closely associated with genomic instability [97], DDR [98], and age-related decline in fitness [99]. Most importantly, telomere dysfunction during aging can amplify and drive other aging mechanisms and the progeria syndrome.

#### Relationship between telomere and telomerase dysfunction and aging

**Fig. 4:**
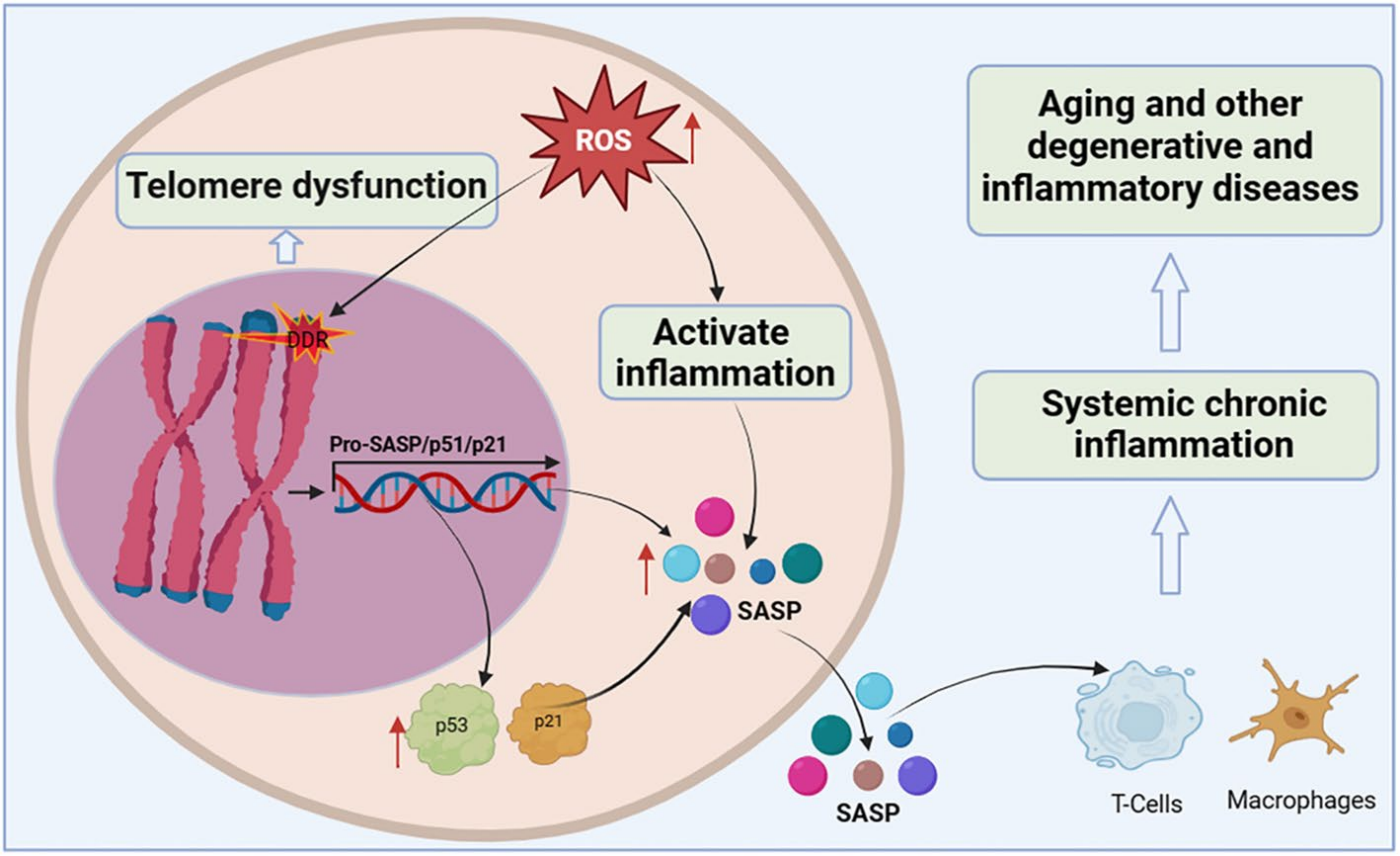
Telomere dysfunction activates DDR to drive cellular senescence. ROS induce telomere sequence damage, leading to telomere shortening and decapitation, triggering DDR, inducing the overexpression of cell cycle inhibition markers p53 and p21, and accelerating cell senescence. Senescent cells secrete SASP, which alter extracellular matrix composition, recruit and enhance T cells and macrophages, which can spread the aging phenotype to surrounding cells, thus promoting systemic chronic inflammation and inflammation-related diseases

Organismal cellular telomere reserves are limited, and the loss of telomere function is closely associated with age-related adaptive decline [99–101] (Fig. 4). Excellent telomere and telomerase structures are essential for ensuring the normal physiological function of mothers and offspring, and their integrity has a certain genetic intergenerational effect [102, 103]. Mice with knockout of TERT that are crossbred in successive generations, the telomeres of the offspring gradually shorten, finally developing telomere dysfunction in the third generation [104]. Additionally, low telomerase levels and continued tissue turnover lead to decades of progressive telomere attrition in the progenitor cells of highly proliferative tissues, including the haematopoietic system, gastrointestinal tract, and skin [10, 11]. Excessive telomere attrition ultimately triggers DDR such as cell cycle arrest [105], apoptosis [106, 107], differentiation disorders [108] and senescence [109]. Notably, as the aging process progresses, hypoproliferative tissues, including the heart, brain, and liver, may suffer from the effects of reactive oxygen species (ROS), which further induce telomere sequence damage, telomere attrition, and uncapping [86, 110]. Thus, the aforementioned telomere properties make them a focal point in the biology of aging.

Shortening of telomeres to a critical length leads to replicative cellular senescence [86, 111–113]. Chromosomal telomeres gradually shorten as DNA replicates. When telomeres reach a critical length, they cannot bind enough telomere-covering proteins and are perceived as exposed DNA ends [114]. One or a few very short telomeres are sufficient to trigger the DNA damage response and induce overexpression of the cell cycle inhibitory markers p53 and p21, thereby forcibly inhibiting cell proliferation [115]. Accumulated senescent cells secrete a complex set of pro-inflammatory cytokines, termed the senescence-associated secretory phenotype (SASP), including interleukins, interleukin chemokines, proteases, and growth factors. The SASP alters the composition of the extracellular matrix and propagates the senescent phenotype to surrounding cells, leading to systemic chronic inflammation [116]. Interestingly, persistent telomere cohesion protected aged cells from premature senescence [117]. Therefore, telomere dysfunction-associated DNA damage response signalling events are key determinants of cell fate and organismal aging.

In summary, telomeres and telomerase play important roles in the core mechanisms that drive aging and many major human diseases. However, many knowledge gaps remain, such as the elucidation of the mechanisms regulating telomerase expression and activity, the non-canonical function of TERT, and the interactions between telomere dysfunction, inflammation, fibrosis, and degenerative disease. Therefore, there is an urgent need to develop telomerase activators for the treatment of aging and age-related diseases to prevent and treat fatal diseases caused by telomere shortening by rescuing telomeres and telomerase damage.

### Aging and mitochondrial dysfunction

Mitochondria are the only organelles that retain their own genome and transcriptional and translational machinery, and are important cellular organelles for cellular energy conversion and signalling. The functional integrity of mitochondria is affected by intramitochondrial protein folding, mitochondrial membrane dynamics, mitosis, and intracellular environmental stress products. One of the classic features of aging is a progressive decline in mitochondrial activity and stress resilience. Mitochondrial dysfunction is closely associated with aging and age-related metabolic diseases.

#### Mitochondrial dysregulation by pleiotropic stress pathways

A healthy mitochondrial network generates adenosine triphosphate (ATP) through the tricarboxylic acid cycle (TCA cycle) and oxidative phosphorylation, which maintain the basic energy conversion and information exchange within the cell and are essential for life [118]. Studies have shown that in normal cells, the nuclear gene-encoded transcription factor PCG1NRF1 induces the expression of mitochondrial-encoding genes, which further regulate mitochondrial biogenesis or increase mitochondrial activity to regulate cellular energy metabolism [119]. Conversely, metabolic perturbations of mitochondrial physiology, such as intramitochondrial protein stabilisation stress, energy deficit, and increased ROS production, trigger transcriptional reprogramming of nuclear genes for metabolic adaptation [120]. Notably, nuclear genes encode most of the mitochondrial proteome, whereas only a few protein-coding genes are encoded by the circular mtDNA. Therefore, to ensure protein balance and functional stability of the mitochondria, it is necessary to maintain excellent mitochondrial-nuclear genome-encoded communication channels [121].

 In addition, mitochondria are the main cellular organelles that regulate energy homeostasis in cellular metabolism, and the dynamic balance of small molecules (including adenosine 5’-monophosphate (AMP), nicotinamide adenine dinucleotide (NAD^+^), oxygen, ROS, and TCA cycle components) produced by mitochondria affect the information of mitochondria, nucleus, and other cellular organelles [14]. Specifically, ATP is a sensitive signal of mitochondrial health, and a continuous decrease in intracellular ATP levels increases the relative AMP content and activates the AMP-protein kinase signalling pathway [122]. The activated 5’-AMP-activated protein kinase (AMPK) signalling pathway further regulates key enzymes in other metabolic pathways (including fat and glucose metabolism, mitochondrial dynamics, autophagy, and protein synthesis) through phosphorylation and indirectly restores the energy balance in the mitochondria [123, 124]. Disruption of this mechanism results in various mitochondria-related diseases. Similarly, NAD^+^ is a cofactor for many metabolic reactions and a key factor in sensing the mitochondrial metabolic state and communicating it to other cellular organelles. We will elaborate on the important role of NAD^+^ in the aging process in Sect. 2.4. Oxygen is another small molecule that affects mitochondrial function; low intracellular oxygen levels reduce the ability of mitochondria to generate ATP [125]. Under normal conditions, cells can stabilise the structure of the proline hydroxylase domain of hypoxia-inducible factor-1/2a, limiting the potential impairment of mitochondrial function caused by low oxygen supply. In addition, toxic byproducts, such as ROS generated in mitochondria, can act on mitochondrial permeability pores together with excess Ca^2+^ in mitochondria, resulting in oxidative damage and swelling of the mitochondria, thereby triggering inflammation and affecting mitochondrial function [126]. Small molecules in the TCA cycle, such as acetyl-CoA, α-ketoglutarate, succinic acid, and fumaric acid, are all signalling molecules that characterise the physiological state of mitochondria.

#### Relationship between mitochondrial dysfunction and aging

**Fig. 5:**
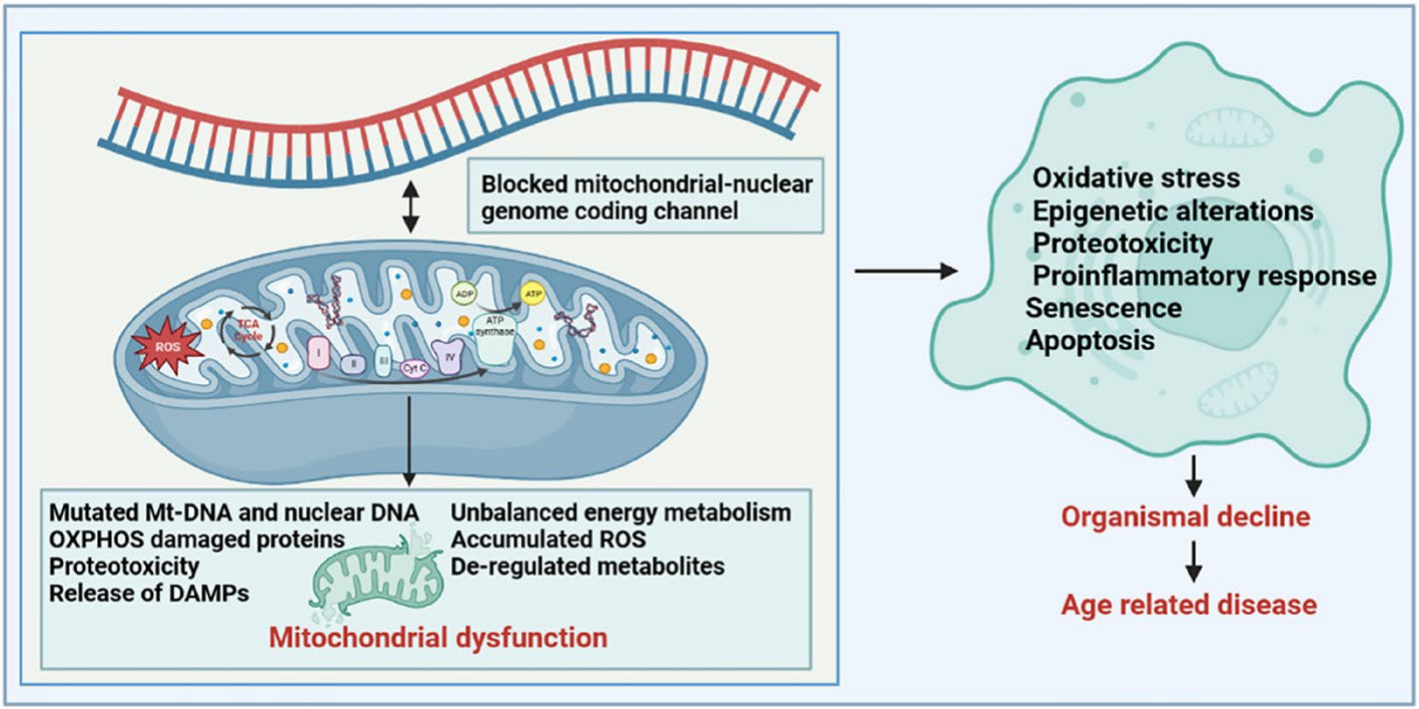
Mitochondrial dysfunction has pleiotropic effects in aging. Inducers such as the accumulation of mtDNA mutations, release of damaged toxic mitochondrial material, the production of mtROS, proteotoxicity, and deregulated metabolites (TCA intermediates, NAD^+^) all contribute to mitochondrial dysfunction. Alterations in mitochondrial function have widespread adverse effects on intracellular homeostasis and lead to systemic organ decline and the development of several age-related diseases through complex signalling mechanisms (involving mitogens, metabolites, etc.)

Mitochondrial dysfunction has pleiotropic effects (Fig. 5). Maintaining healthy and excellent mitochondrial metabolic function is a key factor in ensuring long-term health during the aging process, and the genetic stability of mtDNA and nuclear DNA determines the energy supply capacity of an organism’s tissues throughout life [121]. Unlike mitosis of the nuclear genome, mtDNA can replicate continuously, independently of the cell cycle. Owing to the low repair efficiency of the mtDNA repair system, mutated mtDNA copies accumulate in the cells over time. When the life cycle of an organism enters the later stages of life, heterogeneous mutations generated in both nuclear DNA and mtDNA exceed a certain threshold. These harmful physiological consequences promote the process of aging and age-related diseases, including disturbed glycolipid metabolism, reduced recognition knowledge, and shortened lifespan. Studies have reported that mtDNA mutant mice are more likely to develop signs of premature aging, such as a shortened lifespan, reduced fertility, anaemia, osteoporosis and hearing loss [127]. Notably, perturbation of the mtDNA epigenome has also been implicated in human progeria and disease [119, 128]. The methylation of mtDNA is an important epigenetic modification. During the life cycle, mtDNA methylation is susceptible to environmental interference, endogenous metabolites, and other factors. Studies have found that individuals with reduced methylation in the D-loop region of mtDNA are more likely to develop amyotrophic lateral sclerosis and Parkinson’s disease, whereas those with reduced methylation of Mt-ND1 are more likely to develop Alzheimer’s disease [129] owing to the effect of mitochondrial dysfunction on normal cells. Conversely, senescent cells display changes in mitochondrial morphology, physiology, dynamics, and function. Studies have reported decreased mitochondrial membrane potential, increased proton leakage, and ROS production in senescent cells, further reducing cellular fatty acid oxidation and disrupting mitochondrial metabolism.

In summary, many factors impair mitochondrial function during the life cycle, among which excessively reduced ATP, NAD^+^ and oxygen levels, excessively accumulated ROS levels, and disrupted TCA cycle small molecules are the major contributors. Correspondingly, mitochondrial dysfunction is mainly reflected in transcriptional and epigenetic regulation caused by mitochondrial stress responses, such as mtDNA mutation, and the induction of other cellular organelle disorders, such as lysosomal storage disorders, impaired mitochondrial removal disorders, endoplasmic reticulum response, and changes in the cytoplasmic microenvironment. Based on the sensitivity of mitochondria to their microenvironment, mitochondrial dysfunction has been identified as an important trigger for aging and aging-related metabolic diseases. However, more research is needed to elucidate the interrelationships between mitochondrial dysfunction, aging, and aging-related diseases, as well as the underlying mechanisms of action, to discover new targets for anti-aging interventions.

### Aging and NAD^+^ loss

Nicotinamide adenine dinucleotide (NAD^+^) is an important cofactor in the nucleus, cytoplasm, and mitochondria [130]. NAD^+^ is involved in the regulation of cell redox reactions and energy metabolism, and its abnormal metabolism can affect cell metabolism, DNA repair, organelle function, immune cell viability, and cell aging [131]. However, aging is accompanied by a gradual decline in NAD^+^ levels in tissues and cells, which accelerates the aging process and increases the prevalence of age-related diseases. Therefore, maintaining NAD^+^ levels in tissue cells is important to alleviate the loss of tissue cell function, stabilise metabolic homeostasis, and promote healthy aging.

#### NAD^+^ regulatory network and its role in cellular processes

NAD^+^ is an important coenzyme in cellular redox reactions and is at the centre of energy metabolism [132]. It is involved in regulating the activity of dehydrogenase in metabolic pathways such as cellular glycolysis, fatty acid oxidation, and L-glutamine metabolism [133]. In these reactions, NAD^+^ receives hydrogen ions, forms its reduced form NADH, transfers the accepted electrons to the electron transport chain, and generates ATP to supply energy to the cell. Conversely, NAD^+^ is phosphorylated to form NADP^+^, which then receives hydrogen ions to form NADPH, a process that protects the reducing anabolic pathways from oxidative stress. Notably, NAD^+^ is also a cofactor and substrate for hundreds of cellular enzymes and is one of the major contributors to maintaining cellular processes and ensuring cellular physiological functions [134]. In the early and middle stages of life, NAD^+^ synthesis, metabolism, and consumption are in a balanced state. Specifically, NAD^+^ is continuously utilised in cells by NAD^+^-consuming enzymes, including NAD^+^ glycohydrolases, NADases (CD38, CD157, and Sarm1), and the protein deacetylase family of Sirtuins and poly ADP-ribose polymerases (PARPs), which participate in a variety of important cellular functions and generate the byproduct nicotinamide (NAM). To maintain intracellular NAD^+^ levels, in the NAM recycling pathway, NAM is converted to NMN by nicotinamide phosphoribose transferase (NAMPT) and further converted to NAD^+^ by nicotinamide mononucleotide adenosyltransferases NMNat1 (nucleus), NMNat2 (cytosolic face of the Golgi apparatus), and NMNat3 (mitochondria) [132]. In addition, NAD^+^ can be synthesised from tryptophan via the kenuridine pathway and from vitamin precursors such as nicotinic acid via the Preiss-Handler pathway. Most tryptophan is metabolised to NAM in the liver and converted to NAD^+^ via the NAM rescue pathway [135]. Thus, the NAM rescue pathway appears to be a major contributor to system-wide NAD^+^ levels.

Under normal circumstances, NAD^+^ is continuously decomposed, synthesised, and recycled to maintain the balance and stability of intracellular NAD^+^ levels [136]. However, studies have found that the balance between NAD^+^ catabolic and anabolic processes is altered during aging, with NAD^+^ degradation rates exceeding the capacity for intracellular NAD^+^ synthesis, or excess NAM being broken down by alternative intracellular metabolic pathways, effectively shifting it away from the NAM rescue pathway and further affecting NAD^+^ levels [137]. Studies have demonstrated that when rodents or humans reach middle age, the level of NAD^+^ in the body is reduced to half that at a young age, which severely impairs cellular energy metabolism and various biological pathways, accelerates the aging process, and increases the incidence of age-related diseases [138].

#### Relationship between NAD^+^ loss and aging

**Fig. 6:**
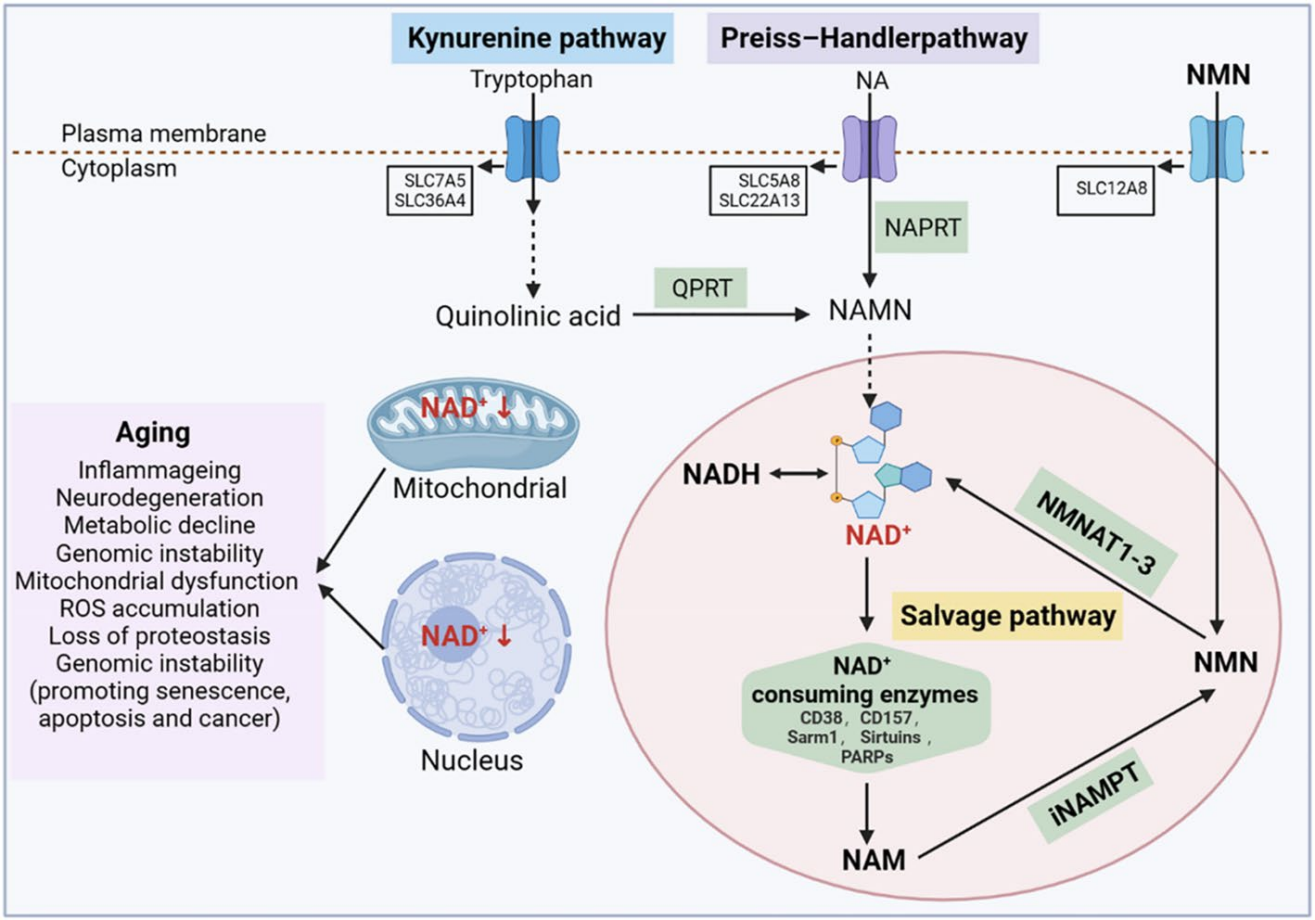
NAD^+^metabolism and its relationship with aging. NAD^+^ levels are maintained by three independent biosynthetic pathways. The kynurenine pathway uses the dietary amino acid tryptophan to produce NAD^+^. Tryptophan enters cells through the transporters SLC7A5 and SLC36A4. In the cell, tryptophan undergoes a series of reactions to form quinolinic acid, which is then converted by the quinolinic acid phosphoribosyl glycosyltransferase (QPRT) into nicotinamide mononucleotide (NAMN), where it converges with the Preiss-Handler pathway. In the Preiss-Handler pathway, niacin (NA) enters cells via SLC5A8 or SLC22A13 transporters, and is catalysed by the nicotinic acid phosphoribosyltransferase (NAPRT) to produce NAMN, which is then converted into NAD^+^ by a series of reactions. The NAD^+^ salvage pathway recycles the nicotinamide (NAM) generated as a by- product of the enzymatic activities of NAD^+^-consuming enzymes (sirtuins, poly (ADP- ribose) polymerases (PARPs) and the NAD^+^ glycohydrolase and cyclic ADP- ribose synthases CD38, CD157 and Sarm1). Intracellular nicotinamide phosphoribotransferase (INAMPT) circulates NAM to nicotinamide mononucleotide (NMN), a portion of which enters the cell via SLC12A8 transporter and is then converted to NAD^+^ by different NMNATs. Decreased levels of NAD^+^ in cells during senescence give rise to a range of problems, including inflammageing, neurodegeneration, genomic instability (promoting senescence, apoptosis, and cancer), mitochondrial dysfunction, ROS accumulation, and loss of proteostasis

NAD^+^ levels are strongly associated with health and aging in both rodents and humans (Fig. 6). In 1937, scientists discovered that low levels of NAD^+^ can lead to symptoms such as dermatitis, diarrhoea, and dementia. NAD^+^ levels gradually decrease during aging, but the mechanism of this reduction is not fully understood. Recent studies have found that aging itself causes inflammation and oxidative stress, which affect the activity of NAMPT, the rate-limiting enzyme of NAD^+^ synthesis, and further affect the activity of downstream NAD^+^-dependent enzymes (including Sirtuins, PARPs, CD38, and CD157) [135]. Notably, Sirtuins, PARPs, and CD38 are the main enzymes that consume NAD^+^, and their content and activity strongly affect intracellular NAD^+^ level [139]. Sirtuins contain seven proteins (Sirtuin1–7) which are a class of NAD^+^-dependent deacetylases. They regulate the activity of various proteins and gene expression by consuming NAD^+^, and has been shown to be an important mechanism for regulating the life span [140]. PARPs activity is an important factor in intracellular NAD^+^ catabolism. PARPs levels increase with age, possibly because DNA damage caused by aging requires PARPs enzymes to participate in repair; however, excessive activation of PARPs promotes the reduction of NAD^+^ levels [141]. In addition, some studies have found that inflammation and SASP accumulation during aging promote the expression and activity of CD38 protein, leading to a partial reduction in NAD^+^ levels and mitochondrial function through the regulation of SIRT3 [142].

At present, many measures are used to inhibit NAD^+^ consumption caused by aging or disease, including supplementation with various NAD^+^ precursors such as nicotinamide mononucleotide (NMN) [143–145] and nicotinamide riboside (NR) [146], activation of NAMPT activity [147], and inhibition of CD38 activity [148]. Notably, Sirtuins, PARPs, and CD38 play active physiological roles in healthy cells. Thus, not every NAD^+^ promotion strategy has a purely beneficial effect on the organism. Increasing NAD^+^ levels by inhibiting PARPs activity reduces the ability of cells to repair DNA damage. Activation of Sirtuins enzyme expression objectively depletes NAD^+^, but also prolongs the lifespan of mice. In conclusion, to gain a more in-depth and comprehensive understanding of the effects of various NAD^+^ promotion strategies, more clinical studies are needed to promote them for practical applications more safely, effectively, and scientifically.

### Aging and disabled macro-autophagy

Autophagy is an indispensable part of cell metabolism that mediates the degradation and elimination of defective cellular components, including damaged nucleic acids, misfolded protein aggregates, abnormal lipids, and organelles, to promote homeostasis, differentiation, development, and survival through lysosomes [149]. Among the molecular phenotypic changes that occur during cellular aging, autophagy disorder has become an important physiological feature and has a causal relationship with aging-related diseases. Therefore, maintenance of an excellent autophagy process is essential for long-term health.

#### Cellular processes involved in autophagy

Autophagy is a highly conserved cell clearance pathway that targets macromolecules and organelles, and the integrity of its biological processes is related to the maintenance of cellular tissue homeostasis. Autophagy can be classified into three main types: macro-autophagy, micro-autophagy, and molecular chaperone-mediated autophagy. Specific target substances of autophagy can be divided into glycophagy and lipidophagy, mitochondrial autophagy, endoplasmic reticulum autophagy, nuclear autophagy, heterologous autophagy, and lysosomal autophagy [150]. These autophagy processes can be summarised as follows: expanded membrane structures (phagocytes) wrap some of the target material, such as defective organelles and misfolded protein aggregates, forming double-membrane sequestering vesicles (autophagosomes). Autophagosomes fuse with lysosomes and release their contents into the lysosomal lumen. The inner membrane of the autophagosome is degraded along with the encapsulated contents, and the resulting macromolecules are released into the cytoplasm for recycling via lysosomal membrane permeases [151]. Autophagy is a tightly regulated pathway that plays an important role in the regulation of basic metabolic functions, enabling cells to remove damaged or harmful components through catabolism and recycling and maintain the dynamic balance of nutrients and energy. Autophagy is also a major protective mechanism that allows cells to survive multiple stress conditions such as nutrient or growth factor deprivation, hypoxia, ROS, DNA damage, or intracellular pathogens [152]. In addition, autophagy is involved in many aging-related pathophysiological processes, such as tumours, metabolic and neurodegenerative diseases, and cardiovascular and pulmonary diseases [153].

#### Relationship between autophagy and aging

The stability or disturbance of autophagy has a causal relationship with health, aging and disease [154]. Increasing evidence indicates that intracellular lysosomal proteolytic function is impaired with aging in various model organisms, which impairs autophagic flux, exacerbates cell damage, and promotes the occurrence of aging-related diseases [155]. In both human clinical studies and rodent models, the expression of autophagy priming-related proteins ATG5-ATG12 and Becn1 decreased with increasing age, whereas the expression of mTOR increased [152]. The fusion rate of neuronal autophagosomes and lysosomes is decreased in aged mice, and neuronal autophagy is reduced, which further leads to the appearance of misfolded, mislocalized, and aggregated proteins in the nervous system and increases the probability of neurodegeneration [156, 157]. These findings suggest a causal relationship between impaired autophagy and aging [158, 159]. This conclusion was confirmed in animal models by manipulating key genes regulating autophagy. Thus, an increase in autophagy caused by heredity, gene mutation, or pharmacological intervention can prolong the life of animals. Studies in *C. elegans* have found that *daf-2* inactivation mutations are dependent on autophagy genes, such as *bec-1*, *lgg-1*, *atg-7*, and *atg-12*, and that this mutation significantly extends the lifespan of *C. elegans* [160]. Enhanced autophagy in aging mice can also activate mitochondrial SIRT3, inhibit oxidative stress and maintain immune memory [161]. Accordingly, researchers have found more damaged autophagy sites in aging model animals, which manifest as reduced autophagosomes and impaired lysosome fusion or degradation ability, accompanied by the accumulation of abnormal organelles or biological macromolecules in the cell, leading to cell dysfunction and even death. This eventually increases the incidence of age-related diseases, such as neurodegenerative, heart, and metabolic diseases [162]. Although the important role of autophagy in inhibiting aging and prolonging lifespan has been widely confirmed, excessive upregulation of autophagy under certain physiological conditions may also cause cell metabolic disorders. For example, the overexpression of Rubicon, a negative regulator of autophagy in aged mice, disrupts adipose metabolism in tissue cells, and the lack of serum/glucocorticoid-regulated kinase-1 (sgk-1) leads to increased mitochondrial permeability and enhanced autophagy, which further leads to reduced environmental adaptability in *C. elegans* and mice [163, 164].

In conclusion, aging is accompanied by a decline in autophagy. Enhancing the autophagic ability in aging model animals is essential for maintaining homeostasis of cell metabolic function, prolonging life span, and improving pathological aging and diseases. Concurrently, autophagy is also one of the important regulators in the aging process. Enhancing or restoring autophagy function to a certain extent is beneficial to the health and longevity of various animal models, whereas dysregulation of autophagy in any direction, whether too low or too high, leads to cell defects and a decline in body function.

### Aging and stem cell exhaustion

Physiologically, the decline in stem cell regenerative ability is closely related to the degree of senescence, which is manifested in the accumulation of global harmful cell metabolites caused by aging, and also impairs the regenerative ability of stem cells. Conversely, stem cell decline is an important cellular driver of a variety of tissue senescence-related pathophysiologies.

#### Main causes of stem cell exhaustion

Stem cells are progenitor cells with the potential for self-replication and multidirectional differentiation. Through self-renewal and differentiation, they can produce mature effector cells, replenish and repair damaged organs, and maintain the health and vitality of the human body; thus, they promote a steady state of continuous organisation throughout the life course [165]. Numerous studies have demonstrated that stem cells play an irreplaceable role in different stages of life. During the growth and development stages, stem cells continue to differentiate into a variety of new cells for growth and development [166]. During adulthood, stem cells replace senescent or damaged cells to maintain normal physiological metabolism of the organism [167]. Notably, throughout the life cycle, stem cells can recognise the signals released by aging damaged cells in the body, localise to the place that needs repair and regeneration, and differentiate into cells at that location to achieve an overall improvement of body functions. However, during the aging process, the proportion of stem cells to total cells gradually decreases. The proportion of mesenchymal stem cell cells in the bone marrow during aging is reportedly 200 times lower than that at birth [168]. Numerous studies have shown that during aging, a series of changes occurs in the tissues and cells of an organism, including increased DNA damage, replication stress, loss of polarity, mitochondrial dysfunction, altered autophagy, and epigenetic disorders, all of which contribute to stem cell aging and exhaustion [20, 21, 169, 170]. In addition, the stem cell microenvironment (also called “Niche”) plays a crucial role in maintaining and regulating stem cell function and tissue homeostasis [171]. During aging, stem cells also accumulate in large quantities as the niche changes, and functional differences between “young and old” stem cells can be more dependent on mechanical differences in the stem cell niche, rather than cell-autonomous age-related changes [172]. This is also risk for stem-cell injection treatments, as the niche itself may need rejuvenation prior to fresh stem cells.

Although stem cells are not affected by replicative senescence, they are still susceptible to damage and accumulate in large quantities during the aging process. Based on the importance of stem cells on the basis of cell lineages, their dysfunction may have a greater impact than that of other cell types [173]. As aging progresses, stem cells tend to accumulate DNA damage, which reduces their ability to regenerate cell lineages, exhibiting age-related loss of organ function and homeostasis, and increasing the incidence of age-related diseases [174]. However, little is known about the cause of this damage or the mechanism by which it leads to a decline in senescent stem cell function. In some cases, DNA damage can lead to stem cell apoptosis, aging, and differentiation, thereby reducing stem cell numbers. Studies have also shown that increased ROS levels in aging mesenchymal stem cells and increased ROS expression in haematopoietic and neural stem cells in mice lead to abnormal cell proliferation, tumour-like changes, and decreased self-replication of stem cells [175]. Similarly, dysregulation of autophagy during aging leads to defects in protein homeostasis, impaired protein folding, and the accumulation of toxic proteins, resulting in cell damage and tissue dysfunction, and stem cells can also be damaged or depleted [176, 177]. Increased mitochondrial DNA point mutations and deletions, along with a shortened lifespan and premature aging, result in decreased nutrient uptake by stem cells. Conversely, enhanced mitochondrial function is accompanied by enhanced stem cell function and tissue regeneration [178]. Epigenetic regulation also plays an important role in the regulation of stem cell function and changes in the epigenome during aging affect the aging process of stem cells [179, 180]. DNA methyltransferases balance self-renewal and differentiation in multiple adult stem cell compartments [181]. Conditional knockout of DNA methyltransferases results in reduced proliferation, abnormal differentiation, and impaired self-renewal of stem cells [182]. In addition, proper histone modification is necessary for stem cell self-renewal, and the activity of histone acetyltransferases is important for maintaining the homeostasis and function of neural stem and progenitor cells [182, 183]. In summary, stem cells are the source cells of organism renewal, and their function is affected by many microenvironmental factors in senescent cells; therefore, stem cell senescence is closely related to the drivers of aging, health, and longevity.

#### Relationship between stem cell exhaustion and aging

During the aging process, a decrease in stem cell number and function is closely related to a decline in tissue function and repair ability. In recent years, with the development of new molecular techniques, such as single-cell transcriptomics, lineage tracing, and clonal analysis, scientists have discovered the commonality and heterogeneity of stem cell senescence across tissues. Particularly, the ability of stem cells to produce offspring is impaired during aging. The number of activated neural stem cells and mature nerve cells (offspring) decreases with age, with older haematopoietic stem cells producing fewer lymphoid cells that are dynamically activated and differentiate more slowly [184]. The fate and behaviour of stem cells in senescent tissues are abnormal, and they may be in a senescent, over-activated, or abnormal differentiation state [185]. In addition, somatic cells in the stem cell pool are more susceptible to mutations and clonal competition during aging, while the heterogeneity of the resting stem cell pool increases, and the ability to produce established offspring decreases [186]. Specific age-related transcriptomic and proteomic markers accumulate in senescent stem cells and induce the infiltration of different types of immune cells into the stem cell microenvironment. Researchers have also found that clonal expansion of T-and B-cell infiltrates occurs in the aging brain tissue of mice and humans, and this infiltrate is more pronounced in age-related diseases [187, 188].

In summary, stem cells, as primitive and undifferentiated cells, possess a strong regenerative capacity and are essential for environmental homeostasis and organ regeneration in mammalian tissues. However, the number of stem cells continues to decrease with age, and their ability to self-renew and differentiate decreases, leading to impaired tissue or organ regeneration. Therefore, stem cell senescence is closely associated with aging. Because of the important role of stem cells in maintaining functional homeostasis during aging, they have attracted considerable attention in the fields of disease therapy, regenerative medicine, and new drug development.

### Aging and cellular senescence

Senescent cells in tissues and organs are thought to be essential not only for the aging process but also for the onset of chronic diseases [189]. During aging, cells exposed to metabolic, genotoxic, or oncogene-induced stress undergo a basically irreversible cell cycle arrest called cellular senescence [190]. A major phenotype of senescent cells and how they are thought to promote disease is an increase in inflammatory mediators, mainly cytokines and chemokines, known as the SASP, it causes dynamic equilibrium damage by interfering with stem cell regeneration, tissue and wound repair, and inflammation [191]. As the number of senescent cells increases with age, cell senescence has been associated with several age-related diseases, the elimination of senescent cells with drugs for aging may be an effective treatment for several previously untreatable diseases [192].

#### Main causes of cellular senescence

Cell senescence is a kind of cell state caused by stress injury and some physiological processes, which is characterized by irreversible cell cycle arrest, accompanied by secretory features, macromolecular damage and metabolic changes, these functions can depend on each other to jointly drive the aging process [193]. Cell senescence may be an alarmist response to deleterious stimuli or aberrant proliferation, including cell cycle exit quiescence and terminal differentiation. Quiescence is a state of temporary arrest in which proliferation can be restored with appropriate stimulation; terminal differentiation is the acquisition of specific cellular functions, accompanied by persistent cell cycle arrest mediated by pathways distinct from cellular senescence [194]. In senescent cells, the cyclin-dependent kinase (CDK2) inhibitor P21^WAF1/CIP1^(CDKN1A) and the CDK4/6 inhibitor p16^INK4A^ (CDKN2A) accumulate, this accumulation leads to sustained activation of retinoblastoma (RB) family proteins, inhibition of E2F transactivation, and subsequent cell cycle arrest [195]. ARF (an alternative reading frame protein at the P16^INK4A^ gene locus that activates p53) has an important role in regulating cell cycle arrest [196]. In addition, cell cycle arrest is also characterized by defects in ribosome biogenesis and retrotransposon [197]. Senescent cells secrete a number of cytokines, including proinflammatory cytokine and chemokines, growth regulators, angiogenic factors and matrix metalloproteinase, collectively known as the SASP or the senescence information secretory group. SASP has been recognized as a marker of senescent cells and mediates many pathophysiological effects [198]. In addition, DNA damage, telomere depletion, epigenetic changes, protein damage, lipid damage, dysfunction of mitochondria and lysosomes, ROS and inflammation are all important inducers of cell senescence [199].

#### Relationship between cellular senescence and aging

Aging is a complex biological process, which is closely related to cell function [200]. Over the past few decades, a growing body of research has found that cellular senescence is a key driver of many age-related diseases. Excessive accumulation of senescent cells may lead to many chronic diseases and accelerate organ aging. This process not only affects health, but also promotes mutual cellular and physical aging.

Senescent cells exhibit abnormally high levels of damage accumulation, including DNA damage, telomere dysfunction, mitochondrial dysfunction and ROS accumulation [201]. Elimination of senescent cells reduces the number of cells with the highest degree of damage [191, 198]. Therefore, treatment methods that improve or delay cellular senescence characteristics are important strategies for delaying aging. Studies found that after clearance of senescent cells, lower levels of telomere-associated foci were detected in aortic epithelial and hepatocytes of aging mice [202, 203]. Similarly, after genetic or pharmacological clearance of senescent cells in mice with chemotherapy-or whole-body irradiation-induced senescence, a reduction in cells bearing persistent DNA damage was observed [204]. Notably, after clearance of senescent cells, the above evidence relates only to DNA damage, and no data currently provide insights into protein or lipid damage or other senescence-associated phenotypes after clearance of senescent cells.

A new class of drugs, “Senolytics,” eliminate senescent cells by inhibiting a targeted pathway that ultimately damages cell apoptosis [205]. The senolytic approach aims to selectively eliminate senescent cells, with a pioneering study showing that about 30% of senescent cells are cleared and that heart, kidney and adipose tissue function is improved [206, 207]. Subsequent research has focused on specific age-related diseases such as frailty, Idiopathic pulmonary fibrosis, arteriosclerosis, osteoporosis, liver steatosis, and osteoarthritis, in these cases senescent cell clearance has been shown to be beneficial [208]. Notably, elimination of senescent cells has been shown to alleviate age-related diseases, but not necessarily successfully delay aging. Aging is generally considered a negative phenomenon, but in the context of disease-free aging, senescent cells can retain at least part of their pre-senescent phenotype and function [209]. The elimination of senescent cells will also result in the filling of the empty space by new cells, which requires the proliferation of stem cells or other resident cells, which may lead to the depletion of their regenerative potential and replication of senescence [198]. Cellular senescence appears to be a trade-off between tissue function and the risk associated with injury accumulation. Therefore, the therapeutic strategy of intermittent short-term clearance of senescent cells provides a new perspective to solve this problem. In summary, the rate at which cellular damage accumulates determines the rate at which cells senescence, and implies the rate at which the aging process occurs in a disease/healthy state. It is reasonable to understand that proper elimination of senescent cells may be an effective strategy to control aging-related diseases and delay aging.

### Other possible causes of aging

Aging is a complex biological process characterised by age-related adaptive decline and a decline in organic physiological systems and metabolic pathways. In addition to the major contributors to aging that we have reviewed, factors such as inflammation, loss of epigenetic information, resurrection of endogenous retroviruses, loss of protein balance, deregulated nutrient sensing, altered intercellular communication, and tissue dysbiosis are also important drivers of accelerated aging.

Many studies have demonstrated that chronic inflammatory response activates the nuclear factor (NF)-κB signalling pathway, a key intracellular signalling pathway for inflammation, which then influences the fate of tissue cells towards senescence by regulating the downstream mechanistic target of rapamycin (mTOR) pathway, insulin and insulin-like growth factor pathways, the AMPK, Sirtuin, forkhead box O families, and p53-related pathways [12, 210–213]. The loss of epigenetic information is also an important cause of aging in mammals. In yeast, epigenetic information is lost over time due to the relocalization of chromatin-modifying proteins to DNA breaks, causing cells to lose their identity, a hallmark of yeast aging [214]. It was reported the act of faithful DNA repair advances aging at physiological, cognitive, and molecular levels, including erosion of the epigenetic landscape, cellular exdifferentiation, senescence, and advancement of the DNA methylation clock, which can be reversed by OSK-mediated rejuvenation [215]. The latest research has found that the resurrection of endogenous retroviruses is a hallmark and driving force of cellular senescence and tissue aging. Activation of endogenous retroviruses has been observed in organs of elderly primates and mice, as well as in human tissues and serum of elderly individuals. Their inhibition alleviates cellular aging and tissue degradation, and to some extent, alleviates the aging of the body [216].

In addition, a decline in the activity of the protein-folding chaperone network and loss of intracellular protein balance are important markers of aging [217]. Decreased Sirtuin1 and HSP70 levels in senescent cells impair protein homeostasis and heat shock response [22, 218]. A growing body of literature has also shown that the ubiquitin-proteasome system is the main non-lysosomal pathway by which cells control protein degradation, either by promoting central lifespan regulators or by aberrant folding and degradation of damaged proteins, and it plays a potential role in regulating the aging process [219, 220]. Aging-induced telomere shortening, mitochondrial dysfunction, and DNA damage affect cellular nutrient perception [221–223]. Aging is also associated with progressive changes in cell-to-cell communication, including factors in the blood-borne system that promote aging or prolong life, interaction of different communication systems between cells, and interference of two-way extracellular matrix communication during aging [223]. Among the blood-derived factors with pro-aging effects, chemokines CCL11, eosinophil granulocyte, and inflammation-related protein b2-microglobulin can reduce neurogenesis, IL-6 and transforming growth factor β can inhibit the haematopoietic system, and complement factor C1q can affect muscle repair [25, 26]. Notably, these blood-derived factors are secreted in the context of SASP, and may contribute to the “infectious” aging phenomenon. Moreover, it has been demonstrated that the anti-aging blood transmissible factors in the blood of young mice can effectively restore the renewal and repair ability of old mice, and reduce the expression of age-related genes [224]. Cell-to-cell communication also involves short-term从 n extracellular molecules, including ROS, nitric oxide, nucleic acids, prostaglandins, and other lipophilic molecules [25]. The interactions between soluble factors released from different tissues may also play a role in pro- or anti-aging effects during the aging process. In essence, all of the above-mentioned causes of senescence can lead to dynamic equilibrium disorder within the cell, thus providing a stable proliferative arrest response to various stressors -- cellular senescence. Although senescence promotes programming during development and wound healing, it also limits tumor initiation. However, dynamic equilibrium disorders within senescent cells and their production of large amounts of SASP will induce an inflammatory state that triggers local and systemic inflammation and tissue damage. The pathological accumulation of senescent cells is also associated with a range of diseases and age-related diseases across the organ system. In preclinical and clinical models of aging and chronic diseases, therapeutic approaches that induce apoptosis of senescent cells or inhibit the senescence-associated secretory phenotype have been shown to be pharmacological targets for delaying systemic aging in the body.

Changes in the gut microflora during aging have also attracted great interest from scientists. The gut microbiota is involved in many physiological processes, such as digestion and absorption of nutrients, protection from pathogens, and production of essential metabolites, including vitamins, amino acid derivatives, secondary bile acids, and short-chain fatty acids [225]. The gut microbiota also signals to peripheral and central nervous system organs and other distant organs and has a strong impact on the overall maintenance of host health [226]. The interruption of the two-way communication between bacteria and the host leads to biological disorders and causes various pathological conditions, such as obesity, type 2 diabetes, ulcerative colitis, neurological disease, cardiovascular disease, and cancer [28, 227]. In addition, vitamin D and magnesium deficiency is associated with aging-related diseases [228]. Vitamin D aids in magnesium absorption, and magnesium helps in the synthesis and activation of vitamin D in the body [229]. This interaction reduces the formation of age-related insoluble proteins, and a deficiency of either vitamin D or magnesium can affect muscle, bone, nerve and heart health.

In summary, many studies have linked biological processes such as telomere dynamics, DNA damage response, mitochondrial dysfunction, NAD^+^ loss, autophagy dysregulation, stem cell exhaustion, inflammation, loss of protein balance, dysregulation of nutrient sensing, changes in cell-to-cell communication, and dysbiosis to the triggers behind the characteristics of aging. Aberrant perturbations in these biological processes create feedback loops that amplify the aging phenotype, accelerate the aging process, and increase the incidence of age-related diseases. However, in the early stage of development, the above-mentioned physiological characteristics of aging to a certain extent also promote the healthy development of juvenile factors. For example, the activation of nutrient-sensing signals during early development contributes to organ development in adolescents; and the activation of nutrient-sensing signals after aging has a largely pro-aging effect [230, 231]; Low-dose mitochondrial dysfunction can stimulate cells to engage in a beneficial antagonistic response through mitosis [232]; appropriate levels of cellular senescence contribute to inhibition of tumour generation and promote wound healing [233]. Therefore, it is an important prerequisite to understand the physiological characteristics of aging and its molecular mechanism in specific life stages and physiological states.

## Anti-aging strategies

The interaction and mutual promotion between aging triggers, aging phenotypic characteristics, and inherited or acquired age-related diseases have become key hotspots of interest for scientists to explore anti-aging strategies. To date, there have been a wide range of interventions aimed at mitigating aging or aging-related morbidity, including dietary regulation and caloric restriction, improved sleep quality, enhanced physical activity, altered microbiota, and exogenous active molecular interventions targeting specific senescence-promoting molecular targets.

### The role of diet and calorie-restriction in delaying aging

Diet is strongly correlated with longevity and disease development. In rodents and humans, the excessive intake of high-calorie foods increases body fat production, fat storage, and obesity. People with obesity are more likely to develop symptoms such as elevated insulin, blood sugar, cholesterol, and triglyceride levels during aging, and a combination of these factors can activate the aging pathway and accelerate the aging process, leading to disease and death [234]. Studies based on diet regulation and calorie restriction have shown that moderate calorie restriction in mice modestly extends the lifespan and improves metabolic, cerebrovascular, and cognitive function indices [235–237]. Dietary restriction improves cognition and reduces plaque burden in mice with Alzheimer’s disease through a mechanism related to mitochondrial function in hippocampal neurons [238]. Researchers recently found that controlling the intake of nutrients, including total calories and macronutrient balance, had a greater effect on aging and metabolic health than the three commonly used life-extension drugs (metformin, sirolimus, and resveratrol) [239, 240]. A recent study also showed that moderate caloric restriction can reduce the production of acidic and cysteine-rich (SPARC) proteins, which are associated with aging, and extend the healthy lifespan of older people, further confirming that improving the diet is an important way to live a long and healthy life [241].

### The role of sleep quality in delaying aging

Sleep is an important factor in the recovery and improvement of physiological systems, including metabolism, endocrine function, immune response, and brain metabolism. Poor sleep accelerates aging and increases the incidence of age-related diseases, such as cognitive decline, Alzheimer’s disease, haematopoietic stem cell dysfunction, and coronary heart disease. Numerous studies have reported that improving the quantity and quality of sleep can be considered as an anti-aging treatment that can prevent, slow, or even reverse the physical decline and degeneration associated with the aging process [242]. One clinical trial reported a significant association between better quality and quantity of sleep and increased plasma levels of S-Klotho, a gene family known as a senescence suppressor that is overexpressed to prolong life [243]. In addition, researchers followed 411 volunteers for eight years and found that poor sleep quality may be a new modifiable risk factor for coronary heart disease in older adults, independent of traditional cardiovascular risk factors [244]. In human trials, adequate sleep has been found to regulate the epigenome of haematopoietic stem and progenitor cells, inhibit inflammatory output, and maintain clonal diversity to slow the decline in the hematopoietic system [245]. Sleep and health undergo age-related changes throughout life, and the impact of sleep deprivation in older people is particularly important [246–248]. However, to improve sleep quality in the older people and reduce age-related sleep issues, further research is needed to investigate the relevant mechanisms.

### The role of exercise in delaying aging

Physical activity causes a series of integrated physiological responses in many tissues in the entire animal kingdom and has been widely accepted to improve the health of physiological tissues. Exercise is strongly associated with changes in plasma microsecretory factors, such as immunomodulatory cytokines, regulatory T cells in lymphoid organs, and inflammatory monocytes. Increased physical activity in aged mice significantly slowed cognitive aging and neurodegeneration, and these improvements were associated with the reduced expression of neuroinflammatory genes in the hippocampus. Proteomics of plasma from exercising mice has revealed a significant increase in the complement cascade inhibitor rapamycin, which binds to brain endothelial cells and reduces the expression of neuroinflammatory genes in mice with acute encephalitis and Alzheimer’s disease [249]. In addition, exercise can induce hippocampal precursor cell proliferation in aged mice by activating platelets, that is, increasing the systemic levels of the platelet-derived exerkine CXCL4/platelet factor 4 (PF4) ameliorates age-related regenerative and cognitive impairments in a hippocampal neurogenesis-dependent manner [250]. Although the benefits of exercise have been demonstrated in several studies, older people are less likely to exercise if they are physically weak or have poor health status. Based on this, researchers were able to ameliorate age-related neurogenesis and cognitive impairment in the hippocampus in the older people by systematically administering plasma from exercising mice, and the key molecular target of this regulation was identified; exercise increased the concentration of liver-derived glycosylphosphatidylinositol-specific phospholipase D1 in the plasma of aged mice [251]. Similarly, many studies have found that exercise can enhance myocardial contractility [252], improve heart pumping function and heart ejection fraction [253], improve blood supply [254] and oxygen supply [255] in all organ systems of the body, and speed up metabolism [256], prolonging the lifespan of cells, thereby slowing the aging process of organs and skin.

### The role of exogenous active molecules in delaying aging

Lifestyle changes, including calorie restriction, sleep regulation, and exercise, are insufficient to extend the healthy lifespan of older people or prevent age-related diseases. Therefore, many studies have focused on the mechanisms underlying the aging process and explored ways to target the hallmark features of aging. Currently, the most promising mechanisms for preventing senescence include inhibition of the mTORC1 signalling pathway [257], clearance of senescent cells [258], and the use of natural metabolites to rejuvenate stem cells [221]. Therefore, the development of synthetic or natural small-molecule compounds that inhibit these signature features is a promising anti-aging strategy.

To date, many synthetic or natural small-molecule compounds have been reported to have the potential to genetically protect or regulate senescence in one or more model species (Table 1). Among them, studies on synthetic compounds including metformin, klotho, PF4, hyaluronan acid, taurine, acarbose, rapamycin, spermidine, NAD^+^ enhancers, nonsteroidal anti-inflammatory drugs, lithium, reverse transcriptase inhibitors, systemic circulating factors, glucosamine, glycine, and 17α-oestradiol, focused on telomere attrition, DNA damage, mitochondrial dysfunction, NAD^+^ loss, disabled macro-autophagy, stem cell exhaustion, regulation of tissue cell perception of nutrients, cell-to-cell communication, and improved stem cell function. By improving the interactions among the hallmarks of aging, these synthetic compounds can eventually alleviate or even reverse the decline in age-related bodily functions.

Senescent cells cannot continue to divide or die in tissues, and secrete a range of pro-inflammatory factors that may recruit inflammatory cells to reshape the extracellular environment, induce abnormal cell death and fibrosis, and inhibit stem cell function [258]. Senescent cells are also closely related to the pathogenesis of osteoporosis, atherosclerosis, hepatic steatosis, pulmonary fibrosis, and osteoarthritis. Therefore, anti-aging methods targeting senescent cells are also very important anti-aging strategies and can be divided into two categories: senescent cell lytic agents (senolytics), whose role is to clear senescent cells, and compounds that combat the effects of various cytokines secreted by senescent cells. Currently, senolytics class targeting senescent cells and SASP include natural polyphenol extract, kinase inhibitors, BCL-2 family inhibitors, heat shock protein inhibitors, BET family protein degraders, P53 stabilizers, cardiac steroids, PPRα agonists and antibiotics.

**Table 1:** Main anti-aging compounds

**Synthetic and Natural small-molecule compounds**			
**Substance**	**Molecular target**		**References**
Metformin	genomic instability, mitochondrial function, stem cell rejuvenation capacity, epigenetic alterations, autophagy, DNA-damage, and telomere attrition		[259, 260]
klotho	increased the levels of multiple platelet factors in plasma, improve cognitive ability		[261, 262]
platelet factor 4 (PF4)	attenuated age-related hippocampal neuroinflammation, elicited synaptic-plasticity-related molecular changes and improved cognition in aged mice		[263]
Hyaluronan acid	reduced inflammation and oxidative stress, improved gut barrier function		[264]
Taurine	Improved the function of bones, muscles, pancreas, brain, fat, intestines and immune system; prolonged healthy life span		[265, 266]
Acarbose	Ameliorated epigenetic changes, nutrient sensing.		[258, 267, 268]
Rapamycin	Ameliorated proteostasis, mitochondrial dysfunction, nutrient sensing, stem cell dysfunction, intercellular communication, and cellular senescence		[257, 269]
Spermidine	Ameliorated epigenetic changes, proteostasis, mitochondrial dysfunction, stem cell dysfunction, and intercellular communication		[270–272]
NAD^+^enhancers	Ameliorated intercellular communication, mitochondrial dysfunction		[130, 137, 273]
Non- steroidal anti- inflammatory drugs	Ameliorated nutrient sensing and intercellular communication		[274–276]
Lithium	Ameliorated telomere attrition, proteostasis, and mitochondrial dysfunction		[276, 277]
Reverse transcriptase inhibitors	Ameliorated DNA damage		[40, 278]
Systemic circulating factors	Ameliorated epigenetic changes, stem cell dysfunction, and intercellular communication		[279, 280]
Glucosamine	Ameliorated proteostasis, mitochondrial dysfunction, and nutrient sensing		[281, 282]
Glycine	Ameliorated nutrient sensing and intercellular communication		[283, 284]
17α-oestradiol	Ameliorated nutrient sensing and intercellular communication		[258, 285]
**Senolytic**			
**Senolytic class**		**Molecular target**	**References**
Natural polyphenol extract	Quercetin, Piperlongumine and analogs, Fisetin, Curcumin, Procyanidin C1, Gingerenone A	Promote senescent cells apoptosis, intercellular communication, autophagy, metabolic changes	[286–291]
Kinase inhibitors	Dasatinib, Nintedanib, R406		[292, 293]
BCL-2 family inhibitors	ABT-263		[294]
Heat shock protein inhibitors	17-DMAG and other HSP90 inhibitors, 25-hydroxycholesterol		[295–297]
BET family protein degraders	ARV825, I-BET151, I-BET762, JQ1, OTX015, PFI-1JQ1		[298]
P53 stabilizers	RG-7112, USP7 inhibitors P5091 and P22077		[299, 300]
Cardiac steroids	Bufalin, cinobufagin, convallatoxin, digoxin, ouabain, peruvocide, proscillaridin A		[301]
PPRα agonists	Fenofibrate		[302]
Antibiotics	Azithromycin, roxithromycin		[303]

## Other anti-aging strategies

Heterochronic blood exchange models have shown that the blood of aging mice accelerates the aging of tissues and cells in young mice [304, 305]. Based on this, the researchers showed that systemic exposure of aged male mice to a fraction of blood plasma from young mice containing platelets decreased neuroinflammation in the hippocampus at the transcriptional and cellular level and ameliorated hippocampal-dependent cognitive impairments [263]. Despite the growing number of reports about heterochronic blood exchange research, it still faces great challenges and risks in the field of anti-aging, both at the technical level and in the application of safety. In addition, mesenchymal stem cell therapy has been shown to improve frailty and facial skin aging [306]. Intravenous mesenchymal stem cell may be an effective treatment for frailty in the elderly. However, the safety and efficacy of stem cell therapy remain controversial, and more studies are needed to verify it. Studies have found that deliberate cold exposure can enhance the nervous system, as well as injury and speed recovery [307]. Saunas are effective at activating cell longevity and anti-cancer factors through heat, this may be a useful tool for people who are too old to engage in physical activity [308], but its physiological metabolic mechanisms and safety thresholds remain to be further confirmed.

## Conclusions and perspectives

The physiological characteristics of aging summarised in this article gradually accumulate over time and contribute to the aging process. Notably, antagonism of an organism’s response to the characteristics of aging also plays a subtle role in the aging process. When the cumulative damage caused by primary and antagonistic markers is no longer compensated for by the complex markers of aging, it means that the rate of aging is accelerated. Furthermore, senescence also relies on the integration of cell-autonomous and non-cell-autonomous mechanisms, and mechanisms that promote senescence can be transmitted between different types of organs and cells. In a metachronous experiment linking the vasculature of young and old mice, extensively characterised by single-cell transcription levels, a spatiotemporal map of the ability of the young system to rejuvenate the senescent system was confirmed, and vice versa; the factors that promote aging have the ability to accelerate the aging of young cells. This may explain why programmed aging usually affects multiple organs in a nearly synchronous manner, causes a comprehensive systemic decline in physiological function, and is an important factor that induces pathological aging.

In conclusion, aging is a gradual and complex process of decline in physiological function, and experiments in animal models have shown that certain interventions may not only extend lifespan, but also increase healthy longevity. However, in *vitro* models, tissue culture studies, and in *vivo* animal models, which are ultimately translated into human studies, are complex and diverse, and only a few models can be used to investigate these differences. There are also significant differences between physiological and pathological aging, and the scientific problem of slowing down aging and extending the healthy lifespan of humans involves a number of challenges, including inadequate regulation, barriers to clinical validation, failure to identify more biomarkers of human aging, and the unknown challenges of introducing new interventions to the market. It is gratifying that years of basic research in the anti-aging field have laid the foundation for explosive biotechnology and industrial applications. In a recent report, researchers used a “visual genetic circuit” to control two pathways of yeast aging, alternative approaches including the lysine deacetylase Sir2-related pathway and the haeme-activating protein (HAP)-related pathway, successfully extending yeast lifespan by 82% [309]. Hence, using modern biological techniques, including genetic manipulation or cell-based therapies with broad implementation prospects, to focus on the discovery of physiological mechanisms and interventions underlying the aging process will greatly advance anti-aging research, delay human aging to the maximum extent, maintain human physiological functions in later years, and mitigate the surge in age-related chronic diseases.

## Data Availability

No datasets were generated or analysed during the current study.

## Abbreviations

NAD^+^: Nicotinamide adenine dinucleotide
DDR: DNA damage response
TERT: Telomerase reverse transcriptase
TERC: Telomerase RNA component
ROS: Reactive oxygen species
SASP: Senescence-associated secretory phenotype
ATP: Adenosine triphosphate
AMP: Adenosine 5’-monophosphate
TCA: Tricarboxylic acid
NAM: Nicotinamide
NAMPT: Nicotinamide phosphoribose transferase
PARPs: Poly ADP-ribose polymerases
AMPK: 5'-AMP-activated protein kinase
PF4: Platelet factor 4
